# A Joint Learning Program for Licensed Nurses and Caregivers on End-of-Life Care in the Community

**DOI:** 10.1093/geroni/igaf122.753

**Published:** 2025-12-31

**Authors:** Yuka Sumikawa, Hanako Numata, Akari Maeda, Noriko Yamamoto-Mitani

**Affiliations:** University of Tokyo, Bunkyo-City, Tokyo, Japan; University of Tokyo, University of Tokyo, Tokyo, Japan; The University of Tokyo Global Nursing Research Center, Tokyo, Tokyo, Japan; The University of Tokyo, Bunkyo, Tokyo, Japan

## Abstract

While hospital deaths have long been the norm in Japan, the super-aging society is also a time of multiple deaths, and many people are now dying outside of medical facilities. In particular, many people who die from diseases other than cancer often have significantly impaired ADLs by the time of death and require care. However, current training programs for caregivers do not include education on end-of-life care, and many caregivers face difficulties in providing end-of-life care. On the other hand, most nurses who work outside of hospitals have experience in end-of-life care, but most of their experience is in hospitals and they are not used to work with non-medical professionals such as formal caregivers. Therefore, we have developed an educational program on end-of-life care, in which nurses and caregivers can learn about end-of-life care together. The concept was to make the education enjoyable and to enable nurses and caregivers to learn together. The educational program was planned mainly by the researchers, incorporating the opinions of nurses and caregivers in the field. An e-learning program was created in which a drama of end-of-life care was created to enhance their empathy for the patients, and lectures by experts were added at key points so that participants could learn specific end-of-life care techniques. Some possible participants shadowed different professions than themselves to learn more about the observations and judgments of other professions. In the future, we are considering evolving the program so that more professions, such as care managers, can learn together.

